# Clinical and Cytogenetic Characterization, and Outcome of Chronic Lymphocytic Leukemia Patients in a Single Tertiary Center in Saudi Arabia

**DOI:** 10.1007/s44228-023-00035-9

**Published:** 2023-04-21

**Authors:** Areej Al Mugairi, Ekremah Alzarea, Abdulaziz Almosa, Feisal Alsomali, Abdulmajeed Alqahtani, Fawaz Alhamied, Faris Albogami, Lubna Al Zajdali, Mohammed AlBalwi, Emad Masaudi, Mohsen Alzahrani, Ayman Al Hijazi, Moussab Damlaj, Ahmed Alaskar

**Affiliations:** 1 Division of Hematopathology, Department of Pathology and Laboratory Medicine, King Abdulaziz Medical City, Ministry of National Guard Health Affairs (MNGHA), Riyadh, Saudi Arabia; 2 King Abdullah International Medical Research Center, KAIMRC, Riyadh, Saudi Arabia; 3 King Saud Bin Abdulaziz University for Health Sciences (KSAU-HS), Riyadh, Saudi Arabia; 4 Division of Molecular Pathology, Department of Pathology and Laboratory Medicine, King Abdulaziz Medical City, Ministry of National Guard Health Affairs (MNGHA), Riyadh, Saudi Arabia; 5 College of Public Health and Health Informatics, King Saud Bin Abdulaziz University for Health Sciences, Riyadh, Saudi Arabia; 6 Divisions of Adult Hematology and Stem Cell Transplant, Department of Oncology, King Abdulaziz Medical City, Ministry of National Guard Health Affairs (MNGHA), P.O. Box 22490, Riyadh, 11426 Saudi Arabia; 7 Saudi Society of Blood and Marrow Transplantation, Riyadh, Saudi Arabia; 8 Sheikh Shakhbout Medical City, Abu Dhabi, United Arab Emirates; 9 Khalifa University, Abu Dhabi, United Arab Emirates

Chronic Lymphocytic Leukemia (CLL) is the most common malignant neoplasm of the lymphatic system in Western countries [1]. The reported age-adjusted incidence rate of CLL in the USA was 4.9 per 10,000 persons per year, with an estimated 4320 deaths in 2021, as per the recent Surveillance, Epidemiology, and End Results (SEER) database [2]. According to the Saudi Cancer Registry Report 1999–2013, CLL represents 9% of all diagnosed leukemias [3]. Fluorescence in situ hybridization (FISH) on interphase nuclei is considered the gold standard for detecting genetic aberrations in CLL, followed by conventional cytogenetics performed on metaphase nuclei [4]. The latter detects chromosomal aberrations in less than half of CLL patients, including translocations. Moreover, recent studies demonstrated its role in detecting complex karyotypes that have been reported in 20% of de novo CLL and associated with unfavorable prognosis [5]. The frontline treatment options depend on several predictive factors, including the patient’s age, clinical stage, comorbidities, and performance status, TP53 aberration (TP53 mutation or deletion 17p), and immunoglobulin heavy chain variant mutation (IGHV) [6, 7]. In the Middle Eastern region, very few published observational studies in CLL had focused on prognostic factors [8]. None of them has described the clinicopathologic features or disease outcome. Therefore, this study aimed to describe CLL’s clinical and biological characteristics in a single tertiary center in Saudi Arabia. Furthermore, we assessed the impact of different prognostic risk factors on the disease outcome.

After due institutional review board (IRB) approval, a total of 80 adult patients were retrospectively identified from King Abdulaziz Medical City in Riyadh electronic health records, including all adult patients (> 18 years old) who have been diagnosed with CLL based on flow cytometry immunophenotyping, from January 2012 till the end of December 2019. Patients’ clinical, biological characteristics, and outcome data were collected by chart review. Flow cytometry, FISH studies for deletion 17p, deletion 13q 27, trisomy 12, deletion 11q, deletion 6q and t(11;14) were performed at diagnosis. TP53 mutation and IGHV mutation tests were not performed due to unavailability.

Patients’ clinical and pathologic characteristics were described using frequencies, while numerical data were presented as median with interquartile range or mean and standard deviation. The Chi-square test was used to compare the outcomes of patients with presence or absence of chromosomal aberrations. Five-year overall survival (OS) was calculated from the date of diagnosis to the date of death or last follow-up. Relapse was defined as evidence of disease progression after achieving complete remission (CR) or partial remission (PR) for ≥ 6 months [7]. Time to relapse was calculated from diagnosis to date of relapse. Survival curves were constructed using the Kaplan–Meier method. We subdivided our patient cohort based on the number of genetic abnormalities detected by FISH/ cytogenetics into three groups: no genetic abnormalities, single genetic abnormality, and two or more genetic abnormalities. The impact of patients’ characteristics, including genetic abnormalities and the number of genetic abnormalities on survival and time to relapse, were evaluated using Cox regression analysis. A test with a *P*-value of less than 0.05 was considered significant. Statistical analyses were performed using SPSS software (SPSS, Chicago, IL).

Among the 80 patients, 52 (65%) were males. Patients’ median age at diagnosis was 65 (range, 35–97) years with median age at treatment initiation of 65.9 years. Out of 55 patients who had available data, the mean time from diagnosis to treatment was around (1.4 ± SD 2.2) years. The mean absolute lymphocyte count at the diagnosis by immunophenotyping studies was 45.8 (range, 1.3–192) × 10^9^/L. Sixty-eight (85%) patients had available FISH studies, and 5 (6%) had successful conventional cytogenetics at diagnosis. Forty-eight (70%) patients had positive FISH studies. Patients’ baseline clinical, laboratory and genetic characteristics are summarized in (Table 1).

**Table 1:** Patients’ clinical characteristics of CLL patient cohort diagnosed 2012–2019

Patients’ laboratory characteristics	Number (%)
Gender	
Male	52 (65)
Female	28 (35)
Age at diagnosis (yrs)	
≤ 60	24 (30)
61–70	27 (34)
71 +	29 (36)
Modified Rai clinical stage	
Low (Rai 0)	15 (20)
Intermediate (Rai I and II)	40 (54)
High (Rai III and IV)	19 (26)
Cytogenetic abnormalities, *n* (%)	
Deletion 13q	27 (41)
Triosomy 12	18 (26.5)
Deletion 11q	9 (14)
Deletion 17p	5 (7)
Deletion 6q	1 (2)
t(11;14)	2 (3)
Number of cytogenetic abnormalities, *n* (%)	
No abnormalities	19 (28)
Single genetic abnormality	34 (50)
≥ 2 genetic abnormalities	15 (22)
CD38+, *n*(%)	8 (11)
B2 microglobulin(> 3.5 mg/L), *n*(%)	27 (56)
Treatment at diagnosis	
Observation	51 (69)
FCR or FR	14 (19)
Chlorambucil and rituxamab	7 (9)
R-CVP	2 (3)
Not known	6 (8)

*FCR* fludarabine and rituximab; *FCR* fludarabine, cyclophosphamide and rituximab; *Chl-R* chlorambucil and rituximab; *R-CVP* rituximab, cyclophosphamide, vincristine and prednisone

The estimated 5-year survival rate was 82%. The median survival time was [12.1 years; (95% CI 9–15)]. At the last follow-up, 58 (73%) patients were alive. Twenty-five (31%) had disease relapse with an estimated 5-year relapse rate of 76% and median relapse time of 7.1 years; (95% CI 4.8–9.4)] (Fig. 1). Seven patients had disease progression and Richter transformation to diffuse large B-cell lymphoma, and one transformed to Hodgkin’s lymphoma at a median time of 60 (1–108) months. All the transformed patients succumbed either because of disease or secondary to treatment complications, except for one patient who has been in remission after completing two cycles of R-EPOCH (rituximab, etoposide, vincristine, cyclophosphamide, doxorubicin) and four cycles of R-CHOP (rituximab plus cyclophosphamide, doxorubicin, vincristine, and prednisone).

**Fig. 1:**
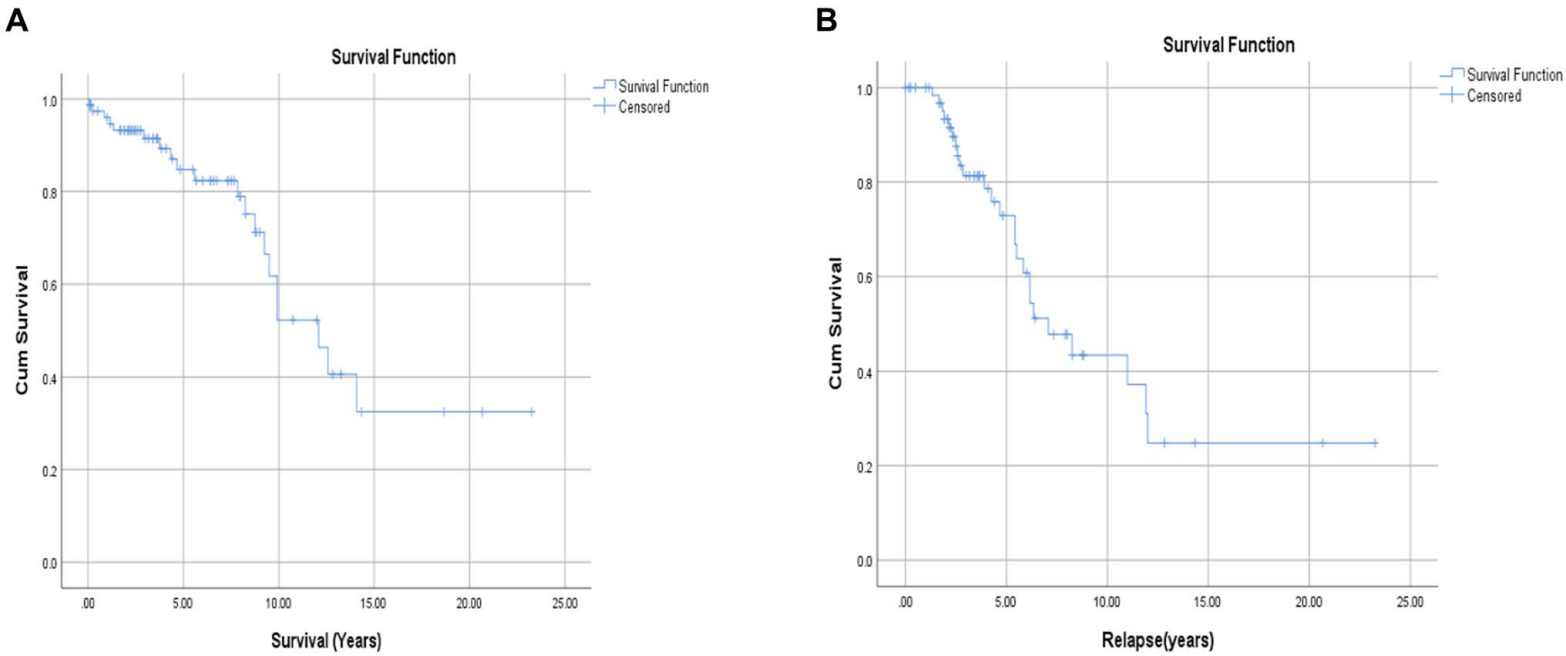
Median survival time and time to relapse for CLL patients diagnosed from 2012–2019

Multivariate Cox regression analysis was conducted to examine the effect of clinical and biological characteristics on overall survival. Patients younger than 60 years (HR = 0.1, *P* = 0.01) and with low clinical stage (Rai 0) (HR = 0.1, *P* = 0.04) had better OS than those older than 60 and with intermediate (Rai I, II) or high clinical stage (Rai III, IV). Moreover, patients with low clinical stage (Rai 0) and younger than 60 years had less tendency for disease progression and relapse but the difference was not significant. Absolute lymphocyte count, CD38 positive status and high B2 micro-globulin did not affect the disease outcome, as shown in Table 2.

**Table 2:** Cox regression analysis of prognostic factors for overall survival and relapse rate for CLL patients diagnosed from 2012–2019

Variable	Survival				Relapse			
	*n* (%)	HR	95% CI	*P* value	*n* (%)	HR	95%CI	*P* value
Age at diagnosis								
≤ 60	3 (12)	0.17	0.04–0.65	0.01	7 (29)	0.52	0.16–1.7	0.28
> 60	8 (29)	0.65	0.24–1.72	0.30	13 (48)	1.5	0.52–4.27	0.44
71+	9 (31)	NA	NA	NA	5 (18)	NA	NA	NA
Gender								
Male	14 (26)	1.59	0.61–4.16	0.34	16 (31)	0.63	0.27–1.44	0.27
Female	6 (21)	NA	NA	NA	9 (33)	NA	NA	NA
Rai clinical stage								
Low	1 (6)	0.12	0.01–0.97	0.04	2 (13)	0.16	0.03–0.83	**0.02**
Intermediate	7 (17)	0.42	0.15–1.15	0.09	14 (35)	0.61	0.23–1.59	0.31
High	8 (42)	NA	NA	NA	6 (33)	NA	NA	NA
Absolute lymphocyte count at diagnosis								
≤ 10 × 10^9^/L	6 (46)	NA	NA	NA	7 (58)	NA	NA	NA
10–30 × 10^9^/L	4 (16)	0.64	0.2–2.3	0.48	7 (28)	0.64	0.21–1.85	0.4
> 30 × 10^9^/L	10 (23)	0.6	0.21–1.66	0.33	11 (26)	0.5	0.19–1.28	0.14
Cytogenetics								
Deletion 11q	3 (33)	NA	0.28–3.49	0.99	8 (88)	1.8	0.77–4.27	0.17
Deletion 13 q	6 (22)	0.51	0.18–1.44	0.21	12 (44)	0.91	0.41–2.00	0.81
Deletion 17p	0 (0)	0.04	0.00–38	0.35	2 (40)	0.57	0.13–2.44	0.44
Trisomy 12	6 (33)	1.33	0.48–3.68	0.58	7 (38)	1.58	0.64–3.88	0.32
Number of cytogenetic abnormalities								
No abnormality	4 (21)	NA	NA	NA	5 (26)	NA	NA	NA
Single abnormality	11 (32)	1.33	0.42–4.19	0.62	11 (33)	0.7	0.23–2.05	0.51
≥ 2 abnormalities	4 (26)	0.68	0.16–2.81	0.59	9 (60)	0.99	0.32–3.02	0.98
CD38 +	2 (25)	1.1	0.24–4.96	0.89	4 (50)	2.56	0.84–7.78	0.09
B2 microglobulin	8 (29)	2.92	0.76–11.0	0.11	10 (37)	0.56	0.21–1.4	0.2

*NA* not applicable

The types of genetic abnormalities frequently described with CLL had no significant effect on the disease outcome, except for 11q deletion. In the subgroup analysis for relapsed patients, out of 9 patients harboring del 11q, 8 (88%) had disease progression and relapse, compared to only 1 (11%) who did not relapse (*P*-value = 0.009) (Table 3). Our data showed that deletion 17p appeared to have a less important negative effect on the risk of relapse. They also disclosed that, whereas there was no significant effect on OS, patients with two or more cytogenetic abnormalities tended to relapse more than those with a single or no genetic abnormality [9 (60%) vs 11(33%) vs 5 (26%); *P* = 0.5] (Table 3).

**Table 3:** Univariate analysis of prognostic factors and disease relapse in CLL study cohort

Variable	Relapse (*N* = 25)		*P*-value
	Yes	No	
Gender, *n* (%)			
Male	16 (31)	35 (69)	0.86
Female	9 (33)	18 (68)	
Age, *n* (%)			
≤ 60	7 (29)	17 (71)	0.37
61–70	13 (48)	14 (52)	**0.02**
≥ 71	5 (19)	22 (82)	
Modified Rai clinical stage, *n* (%)			
Low (Rai 0)	2 (13)	13 (88)	0.19
Intermediate (Rai I and II)	14 (35)	26 (65)	0.90
High (Rai III and IV)	6 (33)	12 (67)	
Number of cytogenetic abnormalities, *n* (%)			
Single	11 (33)	22 (67)	0.59
Dual or multiple	9 (60)	6 (40)	0.05
Absent	5(26)	14 (74)	NA
Trisomy 12, *n* (%)			
Positive	7 (39)	11 (61)	0.87
Negative	18 (37)	31 (63)	
Deletion 11q, *n* (%)			
Positive	8 (88)	1 (11)	**0.009**
Negative	17 (32)	37 (69)	
Deletion 13q, *n* (%)			
Positive	12 (44)	15 (56)	0.40
Negative	13 (34)	25 (66)	
Deletion 17p, *n* (%)			
Positive	2 (40)	3 (60)	0.94
Negative	23 (38)	37 (69)	

The clinical characteristics of our patient cohort are similar to those described on previous studies [9, 10]. In one of the largest prospective USA observational studies (Connect CLL Registry), the median age at diagnosis was 69 years, and the duration from CLL diagnosis to the first line of treatment was approximately 2 years [11]. Comparatively, the median age at diagnosis in our patients was lower (65 years), and with a similar median time to treatment. Moreover, the mean absolute lymphocyte count at diagnosis was higher (45.8 × 10^9^/L) in our patient cohort compared to the one reported by Huang et al. in their large CLL cohort, which may be related to referral center selection bias [10].

The FISH detection rate of cytogenetic abnormalities in our study is slightly lower (70%) than reported by Van Dyke et al. [12], though the distribution of cytogenetic abnormalities is comparable [12]. Nevertheless, it is worth noting that favorable cytogenetic abnormalities (del 13q and trisomy 12q) were more frequent in our study cohort. In contrast, the frequency of 17p deletion was lower than reported in previous studies [10, 12]. Deletion of the long arm of chromosome 11(del 11q) was reported more frequently in our study cohort, and was associated with increased risk of disease relapse, in agreement with previous reports [9, 13].

Moreover, we explored the impact of the number of cytogenetic abnormalities on OS and relapse. We found that patients with more than two cytogenetic abnormalities tended to have more relapse, which is in line with previous study [14].

This is the first report looking at the survival of CLL patients in our region. The estimated 5-year survival rate in our patient cohort is almost identical to that reported in the USA (SEER) database and other reports [10, 15], and it is better than that from a recent report from Taiwan that showed a 5-year survival rate of 60% in Taiwanese CLL patients. The lower OS in that study was related to early treatment and different disease background as explained by its authors [16]. Nevertheless, about a third of our patients had disease relapse. The incidence of Richter transformation was increased (10%) compared to the literature [1, 10]. Interestingly, some of the transformed patients in our cohort had CCND1 rearrangement, trisomy 12, and 13q deletion, but none had 17p deletion. Although we were able to capture all CLL patients diagnosed by flow cytometry test, there are a few limitations related to single center study and non-uniformity of clinical data recording, as is the case in all retrospective studies. Furthermore, the multivariate analysis herein presented is possibly hampered by the small sample size of the cohort.

In conclusion, the clinical and biologic characteristics and the outcome of CLL in our study are comparable to international studies. In addition, ours highlights the infrequent testing for TP53 and IGHV mutations, and emphasizes the importance of implementing these prognostic genetic tests in the real world setting to optimize disease outcome.

## Data Availability

Data can be made available upon request while maintaining patient condidentiality.
